# Differentially Expressed Circular Non-coding RNAs in Atherosclerotic Aortic Vessels and Their Potential Functions in Endothelial Injury

**DOI:** 10.3389/fcvm.2021.657544

**Published:** 2021-07-07

**Authors:** Houwei Li, Xue Liu, Na Sun, Tianshuo Wang, Jia Zhu, Shuang Yang, Xia Song, Ruishuai Wang, Xinhui Wang, Yixiu Zhao, Yan Zhang

**Affiliations:** ^1^Department of Cardiology at the Second Affiliated Hospital of Harbin Medical University, Harbin, China; ^2^Department of Pharmacology (State-Province Key Laboratories of Biomedicine-Pharmaceutics; Key Laboratory of Cardiovascular Medicine Research, Ministry of Education), College of Pharmacy, Harbin Medical University, Harbin, China

**Keywords:** atherosclerosis, circRNA, vascular endothelium, microRNA, ceRNA

## Abstract

**Background:** Circular non-coding RNA (circRNA) has a variety of biological functions. However, the expression profile and potential effects of circRNA on atherosclerosis (AS) and vascular endothelial injury have not been fully elucidated. This study aims to identify the differentially expressed circRNAs in atherosclerotic aortic vessels and predict their potential functions in endothelial injury.

**Method:** ApoE-/- mice were fed with high-fat diet for 12 weeks to induce AS. Atherosclerotic plaques were evaluated by H&E and Masson staining and immunohistochemistry; differentially expressed circRNAs were detected by Arraystar Circular RNA Microarray and verified by RT-PCR; the potential target mircoRNAs of circRNAs were predicted by miRanda, Tarbase, Targetscan and their expression changes were verified by RT-PCR; the potential target genes of mircoRNAs were predicted by Targetscan and verified by Western blot; the signaling pathways that they might annotate or regulate and their potential functions in vascular endothelial injury were predicted by gene enrichment analysis.

**Results:** Fifty two circRNAs were up-regulated more than twice and 47 circRNAs were down-regulated more than 1.5 times in AS aortic vessels. Mmmu_circRNA_36781 and 37699 were up-regulated both in AS aortic vessels and H_2_O_2_-treated mouse aortic endothelial cells (MAECs). The expression of miR-30d-3p and miR-140-3p, the target microRNA of circRNA_37699 and circRNA_36781, were downregulated both in AS vessels and H_2_O_2_-treated MAECs. On the contrary, MKK6 and TP53RK, the potential target gene of miR-140-3p and miR-30d-3p, were upregulated both in AS aortic roots and H_2_O_2_-treated MAECs. Besides, gene enrichment analysis showed that MAPK and PI3K-AKT signaling pathway were the most potential signaling pathways regulated by the differentially expressed circRNAs in atherosclerosis.

**Conclusions:** Mmu_circRNA_36781 (circRNA ABCA1) and 37699 (circRNA KHDRBS1) were significantly up-regulated in AS aortic vessels and H_2_O_2_-treated MAECs. They have potential regulatory effects on atherosclerosis and vascular endothelial injury by targeting miR-30d-3p-TP53RK and miR-140-3p-MKK6 axis and their downstream signaling pathways.

## Introduction

Atherosclerosis is a pathological change of blood vessels characterized with narrowed or blocked vascular lumen by cholesterol or fat deposited under vascular endothelium, thereby causing serious cardiovascular events (1). Atherosclerosis is the most dangerous stimulator of cardiovascular and cerebrovascular diseases. There are many contributors to atherosclerosis, such as hyperlipidemia, oxidative stress, hypoxia. These harmful stimuli impair the function of vascular endothelium and destroy the integrity of the vascular endothelium, leading to lipid deposition and plaque formation (2). Lipid metabolism disorder is the most important predisposing cause of atherosclerosis (3). Excessive circulating low density lipoprotein cholesterol (LDL-C) accumulates in vascular endothelial cells and damages the integrity of endothelium (4). Besides, LDL-C can be oxidized to oxidized-LDL (ox-LDL) by excessive oxygen free radicals in endothelial cells. Ox-LDL deposites in endothelial cells and aggravates vascular endothelial injury further (5). The circulating monocytes and lymphocytes then adhere and invade the endothelial cells, transforming into foam cells and lipid streaks. At the same time, vascular smooth muscle cells proliferate and migrate to endothelium, forming fibrous caps and atherosclerotic plaques (6, 7). Therefore, endothelial injury initiates atherosclerosis and plays a vital role in the pathological progression of atherosclerotic cardiovascular diseases (8). Studies have shown that protecting vascular endothelium could effectively delay the development and deterioration of atherosclerosis (9, 10).

Circular non-coding RNAs (circRNAs) are single stranded RNAs with a closed covalent circular structure. Since circRNAs do not have linear 5′ cap and 3′ ploy A terminals, they are stable and insusceptible to RNA exonuclease. Besides, circRNAs are evolutionarily conserved, and the sequences of circRNA from different species are highly consistent. RNA sequencing and bioinformatic studies have proved that endogenous circRNAs are cell, tissue, and disease specific, so they can be potential candidates for biomarkers and therapeutic targets in the diagnosis and treatment of diseases (11, 12). There are many miRNAs binding sites on circRNAs. The main biological function of circRNAs is serving as microRNA sponges. The complementary binding of circRNA and miRNA reduces the ability of miRNA to down-regulate its target mRNAs, leading to up-regulation of target mRNAs, which is called competitive endogenous RNA (ceRNA) (13–15). Studies have confirmed that circRNAs play important roles in atherosclerosis. CircANRIL regulates the maturation of pre-rRNA, inhibits the proliferation of vascular smooth muscle cells, and reduces atherosclerotic lesions (16). Serum circRNA-284 was significantly elevated and its complementary miR-221 was significantly reduced in patients with carotid artery plaque rupture. In addition, the ratio of circRNA-284 to miRNA-221 was significantly increased in the early stage of carotid plaque rupture. Therefore, circRNA-284 may be a potential biomarker of atherosclerotic plaque stability (17). It was also found that circWDR77 inhibited proliferation of vascular smooth muscle cells by targeting the miR-124 /FGF2 axis; has-circ-003575 was involved in the proliferation of vascular endothelial cells and angiogenesis (18, 19). However, the expression of these circRNAs in animal models of atherosclerosis and their regulatory effect on atherosclerosis have been rarely reported. Although the expression profiles of circRNA, miRNA, and mRNA in carotid artery of atherosclerosis rabbits have been detected by RNA-seq analysis, further laboratory verification has not been carried out (20).

In order to elucidate the role of circRNA at the onset and development of vascular endothelial injury and atherosclerosis, we screened and verified the differentially expressed circRNAs in atherosclerotic vessels and oxidative stress induced endothelial cells. Furthermore, the complementary miRNAs of differentially expressed circRNAs and their downstream mRNAs were predicted by bioinformatics techniques. Meanwhile, the expression of miRNAs and their target genes in atherosclerotic vessels and oxidative stress induced endothelial cells were further validated. Besides, the potential regulatory functions of differentially expressed circRNAs were predicted by KEGG gene enrichment analysis. Our work provides new circRNA targets for the intervention of endothelial injury and atherosclerotic associated cardiovascular diseases.

## Materials and Methods

### Establishment of Atherosclerosis Mice Model

ApoE-/- mice (8 weeks old) were purchased from Beijing Vital River Laboratory Animal Technology Co., Ltd. (Beijing, China). The animal experiments were performed in the science and technology park of Harbin Medical University. All animal protocols were approved by the Ethic Committees of Harbin Medical University (IRB of College of Pharmacy, Harbin Medical University, No. IRB3001619) and were in line with the ARRIVE guidelines and recommendations for the care and use of experimental animals. After adaption for a week, ApoE-/- mice were fed with high-fat diet for 12 weeks to induce atherosclerosis (high-fat diet contained 3% cholesterol, 0.5% sodium cholate, 0.2% propyl thiouracil, 0.5% sugar, 10% lard and 81.3% basal feed). ApoE-/- mice fed with ordinary diet for 12 weeks served as control group.

### Serum Biochemical Analysis

After 12 weeks of feeding on different diets, all mice were anesthetized with pentobarbital sodium after 12 h fasting. Blood from the inner canthus of eyes was collected and serum was separated. Serum level of total cholesterol, triglyceride, LDL-C and high density lipoprotein cholesterol (HDL-C) lipid were determined by automatic biochemical analyzer (Hitachi, Japan) and corresponding biochemical detection kit (MedicalSystem, Ningbo, China).

### Arraystar Circular RNA Microarray

The thoracic aorta was quickly removed from the mouse chest and the aortic root was quickly frozen in the liquid nitrogen. The expression profile of circRNAs in the aortic roots of control and atherosclerosis mice was detected by Arraystar Circular RNA Microarray. These experiments were completed by Kangchen Bio-tech (Shanghai, China).

### Hematoxylin-Eosin Staining

The aortic arches were fixed with 4% paraformaldehyde for 48 h, then embedded with paraffin and cut into 6 μm thick tissue sections. The tissue sections were stained with hematoxylin-eosin (H&E) using a H&E staining kit (Beyotime Biotechnology, Shanghai, China). The nucleus were stained blue, and the cytoplasm and connective tissue were stained red. Representative images were taken by Leica microsystems (Solms, Germany).

### Masson Staining

The aortic arches were fixed with 4% paraformaldehyde for 48 h, then embedded with paraffin and cut into 6 μm thick tissue sections. The tissue sections were stained with Masson using Masson Staining Kit (Solarbio Life Sciences, Beijing, China). Muscle fibers were stained red, collagen fibers were stained blue or green. Representative images were taken by Leica microsystems (Solms, Germany).

### Immunohistochemistry

The aortic arches were fixed with 4% paraformaldehyde for 48 h, then embedded with paraffin and cut into 6 μm thick tissue sections. The tissue sections were fixed on the glass slide pretreated with gelatin and washed in PBS twice with 5 min for each time. Then the tissue sections were treated with 0.1% Triton X-100 penetration solution, washed with PBS for 30 min, and blocked at room temperature for 2 h. Then the sections were incubated with diluted primary antibodies of CD31 (BBI Life Sciences, Shanghai, China) and α-SMA (Boster, Wuhan, China) at 4°C for 24 h and washed in PBS for 6 times with 5 min for each time. Finally, the sections were incubated with diluted secondary antibodies (ZSGB-Bio, Beijing, China) for 2 h, washed with PBS for 6 times with 5 min for each time, and sealed with glycerine sealant. Representative images were taken by Leica microsystems (Solms, Germany).

### Cell Culture

MAECs were purchased from Jining Shiye (Shanghai, China). Cells were cultured with complete culture medium containing 89% H-DMEM (Thermo Scientific, Boston, USA), 10% FBS (Thermo Scientific, Boston, USA) and 1% penicillin-streptomycin and cultured in the incubator containing 95% air and 5% CO_2_. When the cells fuse to 80–90%, subculture can be carried out by digesting cells into single cell suspension using 0.25% trypsin-0.53mM EDTA (Thermo Scientific, Boston, USA). Then cell suspension was cultured in 12-well plates for further experiments.

### RT-PCR

The aortic root tissue or MAECs were treated with 1 mL Trizol (Invitrogen, Boston, USA) and grinded with electric grinder. Then the sample was added with 200 μl chloroform, shocked severely for 20 s, placed on the ice for 10 min, and centrifuged at 4°C and 13,500 r/min for 15 min. Aqueous extract was separated, mixed with equal volume of isopropyl alcohol and blundered slowly, placed on the ice for 10 min, and centrifuged at 4°C and 13,500 r/min for 15 min. Then the supernatant was discarded and the precipitation was washed with 75% ethanol and centrifuged at 4°C, 10,600 r/min for 15 min. The total RNA sample were dissolved with 20 μl DEPC water and transformed into cDNA after reverse transcription using Revert Aid First Strand cDNA Synthesis Kit (Thermo Scientific, Boston, USA). Then cDNA samples were processed into real time PCR using FastStart Universal SYBR Green Master (ROX) (Roche, Basel, Switzerland) to examine the relative level of circRNAs and miRNAs. The primers of circRNAs and miRNAs were listed in Supplementary Material 5, 6.

### Cell Viability Determination

MAECs were incubated with different concentration of H_2_O_2_ (Tianli Chemical Reagent Co., Tianjin, China) for 6 h to induce endothelial injury. Then cells were incubated with 5 mg/mL MTT (Solarbio, Beijing, China) for 4 h to determine cell viability (21).

### Nitric Oxide Determination

MAECs were incubated with 100, 200 μM H_2_O_2_ for 6 h to induce endothelial injury. Then nitric oxide (NO) content was determined by DAF-FM DA Fluorescent Probe Kit (Beyotime, Shanghai, China) (22).

### ROS Determination

MAECs were incubated with 100, 200 μM H_2_O_2_ for 6 h to induce endothelial injury. Then the content of reactive oxygen species (ROS) content was determined by ROS Fluorescent Probe Kit (Beyotime, Shanghai, China) (21).

### Western Blot Analysis

Western blot analysis was performed according to the previous method (23). Protein extraction should be performed at 4°C. Cells or tissues were lysed with ice-cold protein lysates (RIPA: phosphatase inhibitor: protease: inhibitor = 100:10:1) and the lysed mixture was collected into a tube. The lysed mixture was sonicated and centrifuged at 16,000 g and 4°C for 15 min. The supernatant was collected and the protein concentration was measured. The protein samples were heated in a metal bath at 95°C for 10 min. Then the samples were cooled and subjected to gel electrophoresis. Protein samples were added to the gel wells for separation. After complete separation, protein was transferred to an NC membrane and blocked with 5% skim milk for 2 h to block non-specific bands. Then the NC membrane was washed with PBST on a shaker for 30 min and incubated with CD31(Abcam, Cambridge, England), MKK6 (Cell signaling technology, Danvers, MA, United States), TP53RK (Sigma-Aldrich, United States), β-actin primary antibody (1:1,000) at 4°C overnight. Then the NC membrane was washed with PBST on a shaker for 30 min and incubated with the anti-HRP antibody (1:10,000) for 50 min in the dark at room temperature. Then the NC membrane was washed with TBST on a shaker in the dark for 30 min. The expression of the target protein was detected by an infrared fluorescence scanning system, and the optical density integral value of the protein bands was analyzed by Odyssey 1.3 software.

### Statistical Analysis

Data are presented as means ± SEM and analyzed using *t*-test and one-way ANOVA analysis. *P*-value of <0.05 was regarded as statistically significant.

## Results

### The Expression Spectrum of circRNAs Altered Significantly in the AS Aortic Roots

**Figure 1:**
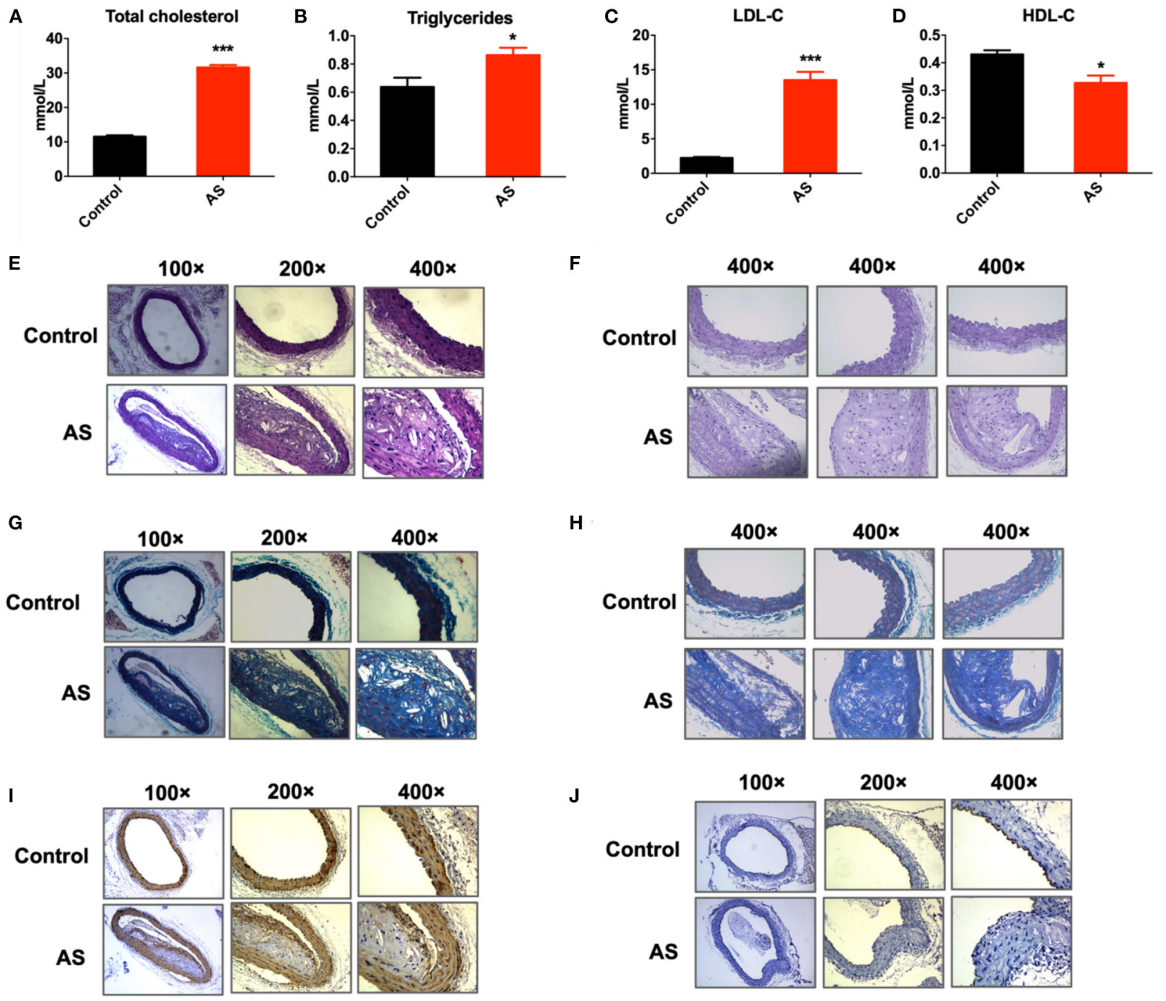
Atherosclerosis mice model was established successfully. **(A–C)** Serum content of total cholesterol, triglycerides, LDL-C of ApoE-/- mice increased significantly after feeding with high fat diet for 12 weeks, *P < 0.05, ***P < 0.001 vs. control, *n* = 6. **(D)** Serum content of HDL-C of ApoE-/- mice decreased significantly after feeding with high fat diet for 12 weeks, *P < 0.05 vs. control, *n* = 6. **(E,F)** H & E staining of aortic arches of control and AS mice showed atherosclerotic plaques and foam cell aggregation in AS vessels. **(G,H)** Masson staining of aortic arches of control and AS mice. **(I)** Expression of α-SMA detected by immunohistochemical staining of aortic arches of control and AS mice. **(J)** Expression of CD31 detected by immunohistochemical staining of aortic arches of control and AS mice; *n* = 3.

ApoE-/- mice were fed with high-fat diet for 12 weeks to induce atherosclerosis (AS). ApoE-/- mice fed with ordinary diet served as control (control). As illustrated in Figures 1A–D, serum total cholesterol, triglycerides, and LDL-C level of AS mice were significantly higher than control; while serum HDL-C level of AS mice were significantly lower than control (*P < 0.05, ***P < 0.001 vs. control, *n* = 3). H&E staining of the AS vessels showed that the arterial intima thickened and lipid deposited under vascular endothelium. Besides, AS vessels showed obvious foam cell aggregation, fat stripe formation, and inflammatory cell aggregation. The intimal surface of AS vessels was covered with atherosclerotic plaques (Figures 1E,F). Masson staining of the AS vessels indicated endothelial hyperplasia and collagen deposition (Figures 1G,H). CD31 immunohistochemical staining of AS vessels suggested that the intimal integrity of the artery was damaged (Figure 1J). α-SMA immunohistochemical staining of AS vessels showed the proliferation and migration of smooth muscle cells to the intima (Figure 1I). In all, the above results suggested that the atherosclerosis model was successfully established in this study.

**Figure 2:**
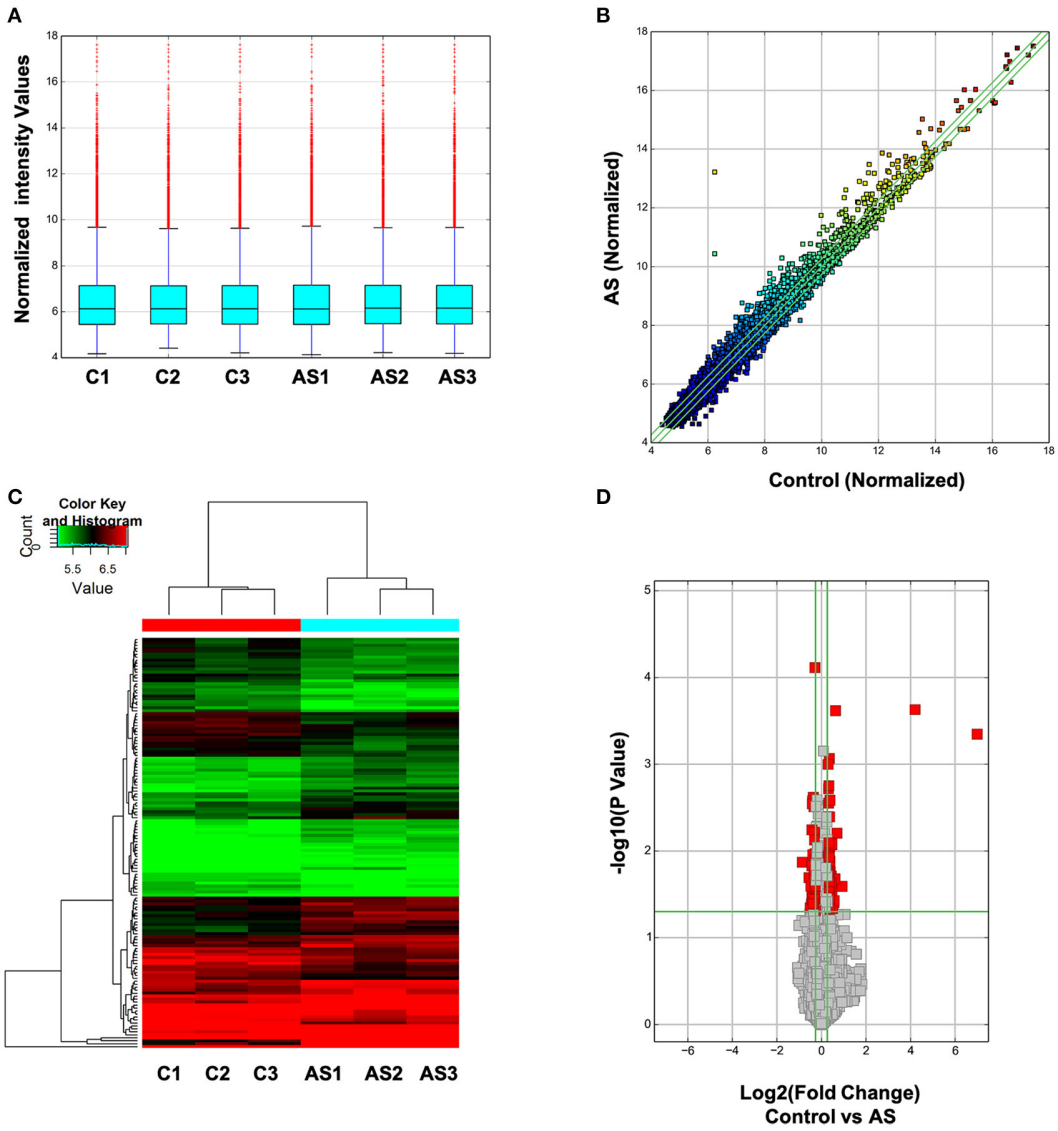
Expression spectrum of circRNAs was screened and analyzed by Arraystar circRNA Microarray Chip. **(A)** Data standardization. **(B)** The overall distribution of chip data. The horizontal and vertical axes represent circRNA expression of different samples respectively. The closer the data get to the center diagonal, the closer the circRNA expressed in two group; while the farther away the data get to the center diagonal, the greater the difference in circRNA expression level between two samples. **(C)** Cluster analysis of differentially expressed circRNA in each sample. Red represents high expressed circRNAs and green represents low expressed circRNAs. **(D)** Differentially expressed circRNA with statistically significant difference between control and AS group.

Next, the expression spectrum of circRNAs in Control and AS vessels was detected by Microarray of Arraystar Circular RNA. As illustrated in Figure 2, after data standardization, the medians of each group of circRNA chip were at the same level and the probes distributed closely, indicating that the quality of chip data was reliable (Figure 2A). Results showed that 52 circRNAs were up-regulated more than twice and 47 circRNAs were down-regulated more than 1.5 times in AS vessels (Figures 2B–D). All differentially expressed circRNAs were listed in Supplementary Materials 1–3.

### Differentially Expressed circRNAs in circRNA Microarray, Aortic Roots of AS Mice and H_2_O_2_-Treated MAECs

**Figure 3:**
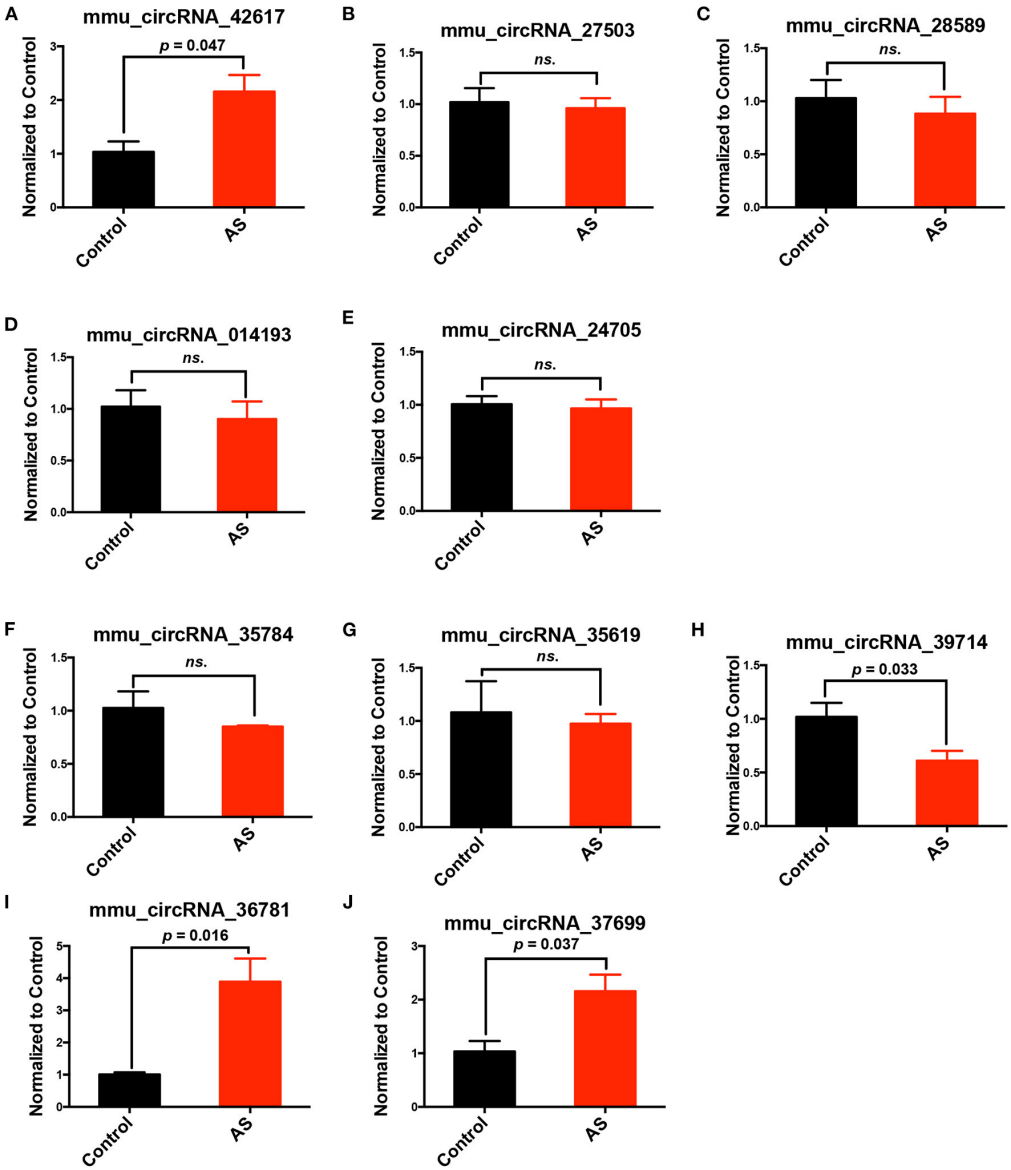
Validation of differentially expressed circRNAs in AS vessels. **(A–E)** Expression of mmu_circRNA_42617, mmu_circRNA_27503, mmu_circRNA_28589, mmu_circRNA_014193, mmu_circRNA_24705 in aortic arches of control and AS mice; **(F–J)** mmu_circRNA_35784, mmu_circRNA_35619, mmu_circRNA_39714, mmu_circRNA_36781,mmu_circRNA_37699 in aortic arches of control and AS mice; *n* = 3. ns, no significant difference.

From all differentially expressed circRNAs, 5 up-regulated and 5 down-regulated circRNAs were selected, according to the significance of differences between two groups and their endogenous expression levels, to validate their expression differences in AS and control aortas using RT-PCR. The selected circRNAs were listed in Table 1. The whole sequences and primer sequence of selected circRNAs were listed in Supplementary Materials 4, 5. Results showed that mmu_circRNA_36781and mmu_circRNA_37699 were significantly up-regulated in AS vessels compared with control. This trend is consistent with the results of chip screening. Besides, mmu_circRNA_42617 and 39714 in AS aortic roots were also significantly altered, but their trends were contrary to the results of microarray screening (Figure 3).

**Table 1:** Selected significantly differentially expressed circRNAs in AS mice.

**circRNA**	**Up or down**	**CircRNA type**	**Gene Symbol**	**Fold change**	***P*-value**
mmu_circRNA_42617	Down	exonic	Csmd1	1.8145739	0.013
mmu_circRNA_27503	Down	exonic	Txndc16	1.8483242	0.063
mmu_circRNA_28589	Down	exonic	Csmd3	1.6626629	0.065
mmu_circRNA_014193	Down	Sense overlapping	Rbm39	1.5368923	0.181
mmu_circRNA_24705	Down	exonic	XLOC_004970	2.1051165	0.220
mmu_circRNA_35784	Up	exonic	Il6ra	1.611438	0.006
mmu_circRNA_35619	Up	exonic	Rapgef2	1.888969	0.025
mmu_circRNA_39714	Up	exonic	Wdr95	2.0716431	0.054
mmu_circRNA_36781	Up	exonic	Abca1	1.7430866	0.092
mmu_circRNA_37699	Up	exonic	Khdrbs1	2.1694076	0.139

Five up-regulated and five down-regulated circRNAs were selected from all differentially expressed circRNAs, according to their expression differences between two groups and their endogenous expression levels, to validate their expression differences further in AS and control vessels using RT-PCR.

**Figure 4:**
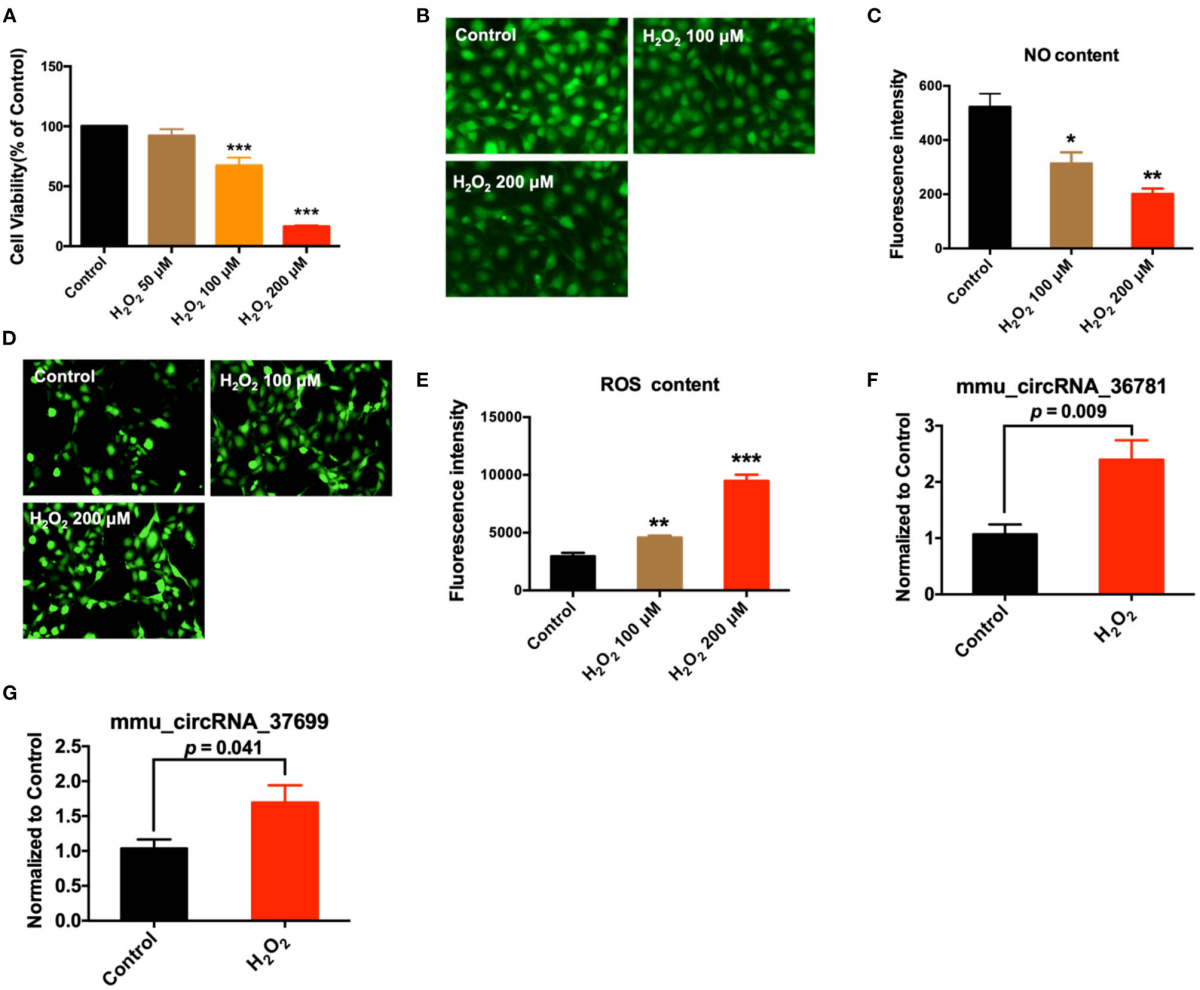
Validation of differentially expressed circRNAs in MAECs induced by H_2_O_2_. **(A)** Effect of H_2_O_2_ on cell viability; ***P < 0.001 vs. Control, *n* = 4. **(B,C)** Effect of H_2_O_2_ on NO concentration of MAECs; 400×, *n* = 3. *P < 0.05, **P < 0.01 vs. Control. **(D,E)** Effect of H_2_O_2_ on ROS concentration of MAECs; 400×, *n* = 3. **P < 0.01, ***P < 0.001 vs. Control. **(F,G)** Expression of mmu_circRNA_36781 and mmu_circRNA_37699 in MAECs induced by 100 μM H_2_O_2_, *n* = 5–6.

In addition, we further verified the expression of mmu_circRNA_36781 and mmu_circRNA_37699 in endothelial injury using oxidative stress injured endothelial cells. MAECs were treated with H_2_O_2_ for 6 h to induce endothelial injury. As shown in Figure 4, H_2_O_2_ significantly decreased cell viability and NO release, increased reactive oxygen species (ROS) content of MAECs (***P < 0.001, Figures 4A–E). Then the expression of mmu_circRNA_36781 and mmu_circRNA_37699 were detected in MAECs pretreated with or without H_2_O_2_. Results showed that both mmu_circRNA_36781 and mmu_circRNA_37699 were significantly up-regulated in H_2_O_2_-treated MAECs (100 μM, 6 h) (Figures 4F,G). The variation trend of mmu_circRNA_36781 and mmu_circRNA_37699 was consistent with the results of microarray and *in vivo* validation.

### Expression of Predicted Complementary microRNAs in H_2_O_2_-Treated MAECs

**Figure 5:**
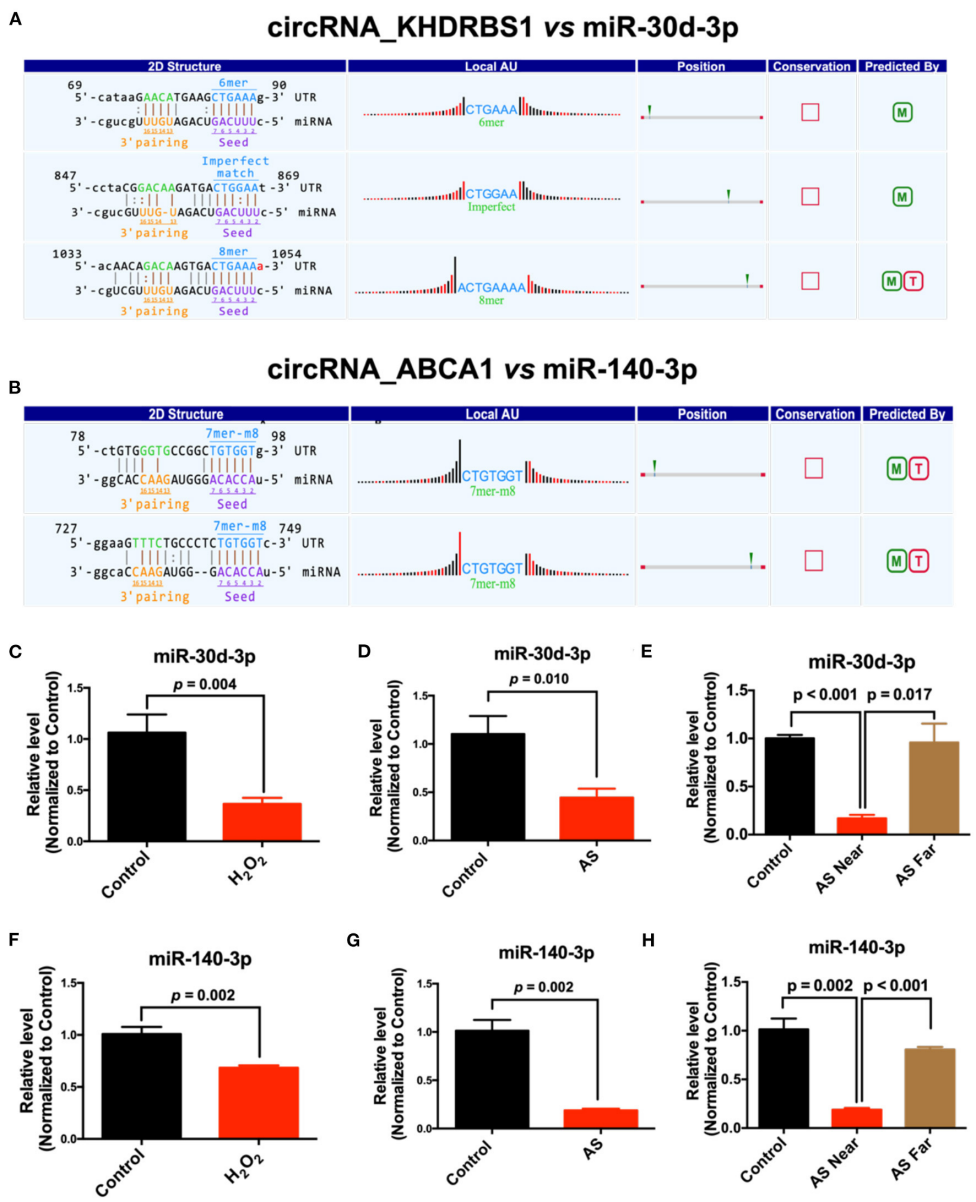
Prediction and validation of complementary microRNAs of differentially expressed circRNAs. **(A)** The binding sites and 2D structure of circRNA KHDRBS1 with miR-30d-3p; **(B)** The binding sites and 2D structure of circRNA ABCA1 with miR-140-3p; **(C–E)** Expression of miR-30d-3p and miR-140-3p in MAECs induced by H_2_O_2_ and AS aorta, *n* = 3–6; **(F–H)** Expression of miR-140-3p in MAECs induced by H_2_O_2_ and AS aorta, *n* = 3–5.

The target microRNAs of differentially expressed circRNAs and their binding sites were predicted using miRanda and TargetScan database. We analyzed these microRNAs and selected those that have the possibility to regulate atherosclerosis and evolutionarily conserved, including, miR-30d-3p and miR-140-3p, which had conserved binding sites with mmu_circRNA_37699 (circRNA KHDRBS1) and mmu_circRNA_36781 (circRNA ABCA1). 2D structure of binding sites was illustrated in Figures 5A,B. Then the expression of predicted complementary microRNAs of above differentially expressed circRNAs were determined in H_2_O_2_ induced MAECs. The primer sequences of miRNAs were listed in Supplementary Material 6. Results showed that miR-30d-3p and miR-140-3p were down-regulated in H_2_O_2_-treated MAECs (Figures 5C,D). In addition, the expression of these miRNAs was also determined in AS vessels. Results showed that miR-30d-3p and miR-140-3p were all significantly down-regulated in AS vessels. The variation trend of miR-30d-3p and miR-140-3p was consistent with the results of *in vitro* study (Figures 5E,F). To further confirm the correlation between miR-30d-3p/miR-140-3p and atherosclerosis, the expressions of miR-30d-3p and miR-140-3p were determined in control aorta (Control), AS aorta near the heart (AS near, susceptible area of atherosclerosis) and AS aorta far away from the heart (AS far, non-susceptible area of atherosclerosis). Results showed that the expressions of miR-30d-3p and miR-140-3p were both significantly downregulated in AS near tissues, while upregulated in control and AS far tissues (Figures 5G,H).

### Expression of Predicted Complementary mRNAs in AS Vessels and H_2_O_2_-Treated MAECs

**Figure 6:**
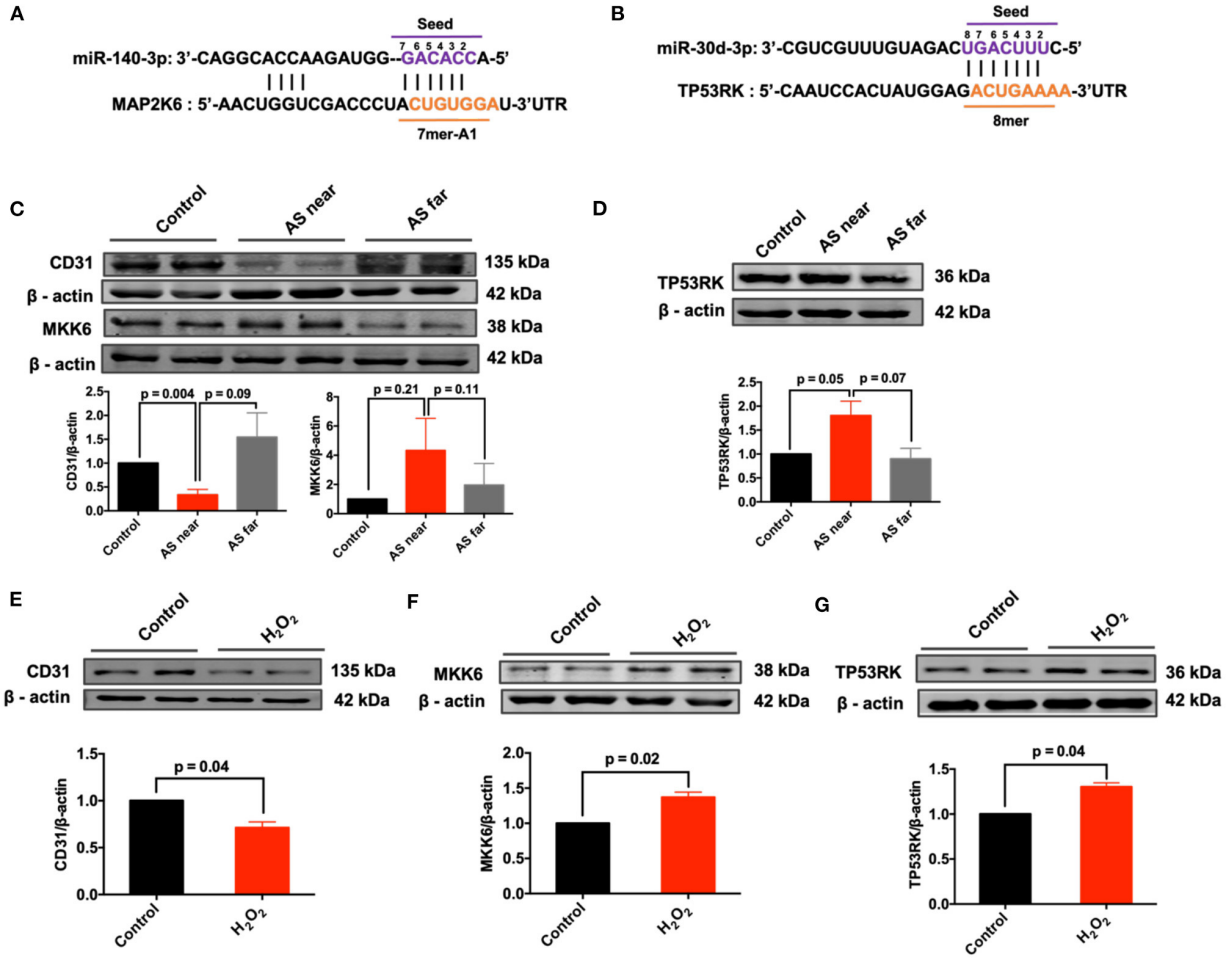
Expression of predicted complementary mRNAs in AS aortas and H_2_O_2_-treated MAECs. **(A)** The binding sites and 2D structure of miR-140-3p with MAP2K6; **(B)** The binding sites and 2D structure of miR-30d-3p with TP53RK; **(C)** Expression of CD31 and MKK6 in control, AS near and AS far aortas, *n* = 3; **(D)** Expression of TP53RK in control, AS near and AS far aortas, *n* = 3; **(E–G)** Expression of CD31, MKK6 and TP53RK in H_2_O_2_-treated MAECs, *n* = 3–4.

Next, the conserved mRNA sequences and their coding genes with complementary binding sites to miR-30d-3p and miR-140-3p were predicted by Target Scan database. The results showed that miR-140-3p and MAP2K6, miR-30d-3p and TP53RK had conservative complementary binding sites (Figures 6A,B). Meanwhile, the protein expressions of MKK6 (protein encoded by MAP2K6) and TP53RK in AS aortas and H_2_O_2_-treated MAECs were detected. Besides, the down-regulation of CD31 expression can be used as a marker of endothelial injury. Results showed that MKK6 and TP53RK were up-regulated in AS aorta near the heart (AS near) and H_2_O_2_-treated vascular endothelial cells, contrary to the expression of miR-140-3p and miR-30d-3p (Figures 6C–G).

### Underlying Target Genes of Differentially Expressed miRNAs and Their Potential Roles in Regulating Atherosclerosis and Vascular Endothelial Injury

**Figure 7:**
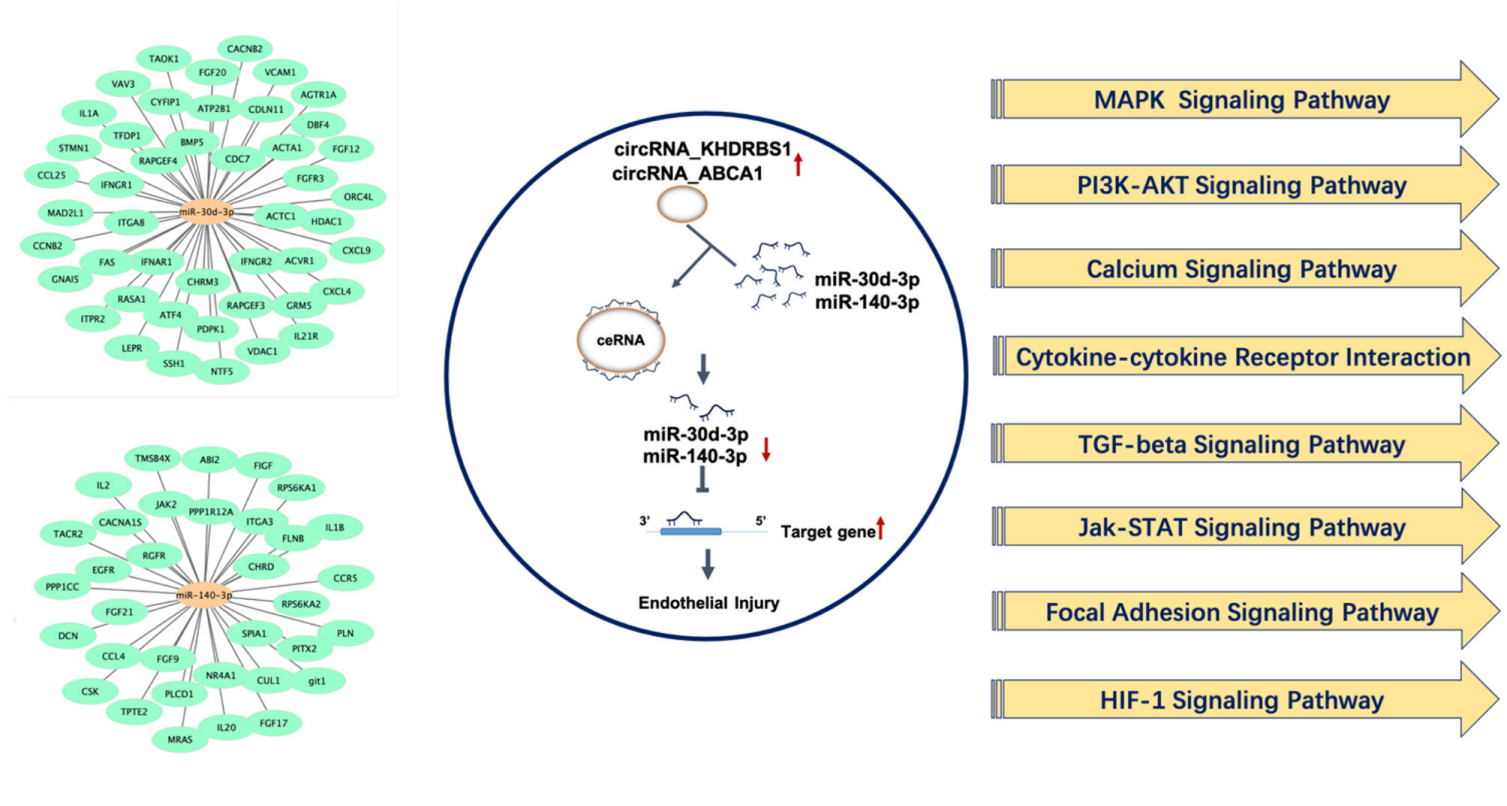
Underlying target genes of differentially expressed circRNAs and their potential function in regulating endothelial injury and atherosclerosis.

On the basis of existing studies and bioinformatic database, target genes of differentially expressed microRNAs which have potential regulatory effects on atherosclerosis and endothelial dysfunction were predicted. Based on the above predicted target genes, the potential functions of circRNAs in atherosclerosis and endothelial injury were predicted by KEGG analysis and summarized in Table 2. Results showed that MAPK and PI3K-Akt signaling pathway were top-ranked signaling pathways. Besides, cytokine-cytokine receptor interaction signaling pathway, calcium signaling, TGF-β signaling pathway, Jak-STAT signaling pathway, focal adhesion signaling pathway, HIF-1signaling pathway may also be potential pathways for differentially expressed circRNAs to regulate the pathological process of endothelial injury and atherosclerosis (Figure 7).

**Table 2:** KEGG pathway analysis.

**Term**	**Gene number**	**Genes**
MAPK signaling pathway	16	FGF9, FGF17, MRAS, CACNB2, FAS, RASA1, IL1A, EGFR, TAOK1, FGF21, FGF20, CACNA1S, ATF4, RPS6KA1, RPS6KA2, STMN1
PI3K-Akt signaling pathway	13	FGF9, FGF17, PDPK1, FGFR3, EGFR, FGF21, FGF20, ATF4, CCNB2, ITGA8, VEGFA, JAK2, IL2
Cytokine-cytokine receptor interaction signaling pathway	13	LEPR, IL21R, CXCL9, CCL4, CCL25, IFNAR1, CCR5, FAS, IFNGR2, IL1A, IFNGR1, IL2, ACVR1
Calcium signaling pathway	8	EGFR, TACR2, CACNA1S, VDAC1, ATP2B1, CHRM3, PLN, PLCD1
TGF-beta signaling pathway	7	DCN, CHRD, CUL1, BMP5, PITX2, ACVR1, TFDP1
Jak-STAT signaling pathway	7	LEPR, IL21R, IL20, JAK2, IFNGR2, IFNGR1, IL2
Focal adhesion signaling pathway	7	EGFR, PPP1CC, PDPK1, ITGA6, CCNB2, ITGA8, PPP1R12A
HIF-1 signaling pathway	5	CDC7, MAD2L1, DBF4, CUL1, TFDP1

Term indicates the name of the signaling pathway; gene number indicates the number of differentially expressed genes that are annotated in the pathway; genes indicated the target genes of differentially expressed microRNAs that are annotated in the pathway.

## Discussion

Atherosclerosis has continued to be prevalent in developing countries in recent years. The pathogenesis of atherosclerosis is complex. Vascular endothelial injury is the initial stage of atherosclerotic plaques formation, which plays a decisive role in the prognosis of atherosclerosis. CircRNAs are endogenous non-coding RNAs with stable circular structure. Studies have shown that circRNAs have regulative effects on vascular endothelial injury, atherosclerosis and related cardiovascular diseases. This study was the first to analyze the differentially expressed circRNAs in atherosclerotic vessels using microarray. We found that circRNA ABCA1 and circRNA KHDRBS1 were upregulated in atherosclerotic vessels and oxidative-stress damaged endothelial cells, which was consistent with the results of microarray. Besides, miR-30d-3p and miR-140-3p with conserved complementary binding sites for circRNA KHDRBS1 and circRNA ABCA1 were down-regulated in atherosclerotic vessels and oxidative-stress damaged endothelial cells, contrary to the changes of corresponding circRNAs. In addition, MKK6 and TP53RK, the potential targets of miR-30d-3p and miR-140-3p, were up-regulated in atherosclerotic vessels and oxidative-stress damaged endothelial cells, further suggesting that circRNA KHDRBS1 and circRNA ABCA1 may regulate vascular endothelial injury and atherosclerosis through the ceRNA mechanism. Other targets and signaling pathways that may be regulated by miR-30d-3p and miR-140-3p were also predicted and analyzed to provide a basis for further studies on the regulation of circRNA KHDRBS1 and circRNA ABCA1 in vascular endothelial injury and atherosclerosis.

CircRNAs have been reported to be involved in a variety of diseases. Current research suggest that circRNAs perform their biological functions through participating in post-transcriptional regulation of genes. Specifically, circRNAs can act as microRNA sponges, competitively inhibiting complementary microRNAs through the ceRNA mechanism and influencing the function of related mRNAs (24). Currently, several studies have reported the potential roles of circRNAs in regulating atherosclerosis, including the regulation of endothelial cell proliferation and angiogenesis by hsa_circRNA_0003575; circANRIL promotes atherosclerosis by modulating ribosomal RNA production and increasing nuclear pressure; reduced circANRIL reduces cell apoptosis, inflammatory factors expression and endothelial damage (16, 19, 25). However, the expression spectrum of circRNAs in atherosclerotic vessels, the relationship between circRNAs and atherosclerosis, and the potential mechanisms of circRNAs regulating atherosclerosis and endothelial injury have not been reported. In this study, we detected and analyzed the differences in circRNA expression profiles between AS and normal vessels. The expression of circRNAs in AS vessels changed significantly compared with normal vessels. Laboratory verification of microarray data showed that mmu_circRNA_36781, mmu_circRNA_37699 in AS vessels were significantly up-regulated and these changes were consistent with the microarray data. Therefore, we screened and verified the differentially expressed circRNAs in atherosclerotic vessels for the first time, providing a basis for further research on the biological functions of circRNAs in regulating atherosclerosis.

Relevant studies have shown that circRNAs regulate downstream target genes by acting as molecular sponges of complementary microRNAs (26). Therefore, we used bioinformatics databases to predict the microRNAs that had specific and conserved binding sites with these differentially expressed circRNAs. Through a comprehensive analysis of interspecies conservatism and binding stability, we selected miR-140-3p and miR-30d-3p as potential microRNA targets. Then, we detected the expression of these microRNAs in AS vessels and H_2_O_2_-treated MAECs. The results showed that miR-140-3p and miR-30d-3p were significantly down-regulated in H_2_O_2_-treated MAECs and AS vessels. Therefore, the variation trend of miR-140-3p and miR-30d-3p was consistent in H_2_O_2_-treated MAECs and AS vessels, contrary to the changes of corresponding circRNAs. Therefore, we speculate that circRNA_ABCA1 and circRNA_KHDRBS1 may regulate vascular endothelial injury by targeting miR-140-3p and miR-30d-3p, respectively.

Current studies on miR-140-3p have shown that miR-140-3p protects against ox-LDL, high glucose and ischemia reperfusion induced endothelial injury, and may have a potential preventive effect on vascular disease (27–29). Recent research has reported that miR-30d-3p can reduce cerebral ischemia reperfusion injury (30). Therefore, miR-30d-3p and miR-140-3p may have important biological functions in vascular disease. Based on the protective potential of miR-140-3p and miR-30d-3p on vascular endothelium, their regulative effects on atherosclerosis and whether they can be used as therapeutic targets for atherosclerosis and vascular endothelial injury require further studies. This study found that circRNA_ABCA1 and circRNA_KHDRBS1 had the potential to regulate miR-140-3p and miR-30d-3p and may be key regulators of miRNA abnormalities during disease. Besides, potential target genes of miR-140-3p and miR-30d-3p associated with atherosclerosis or endothelial injury were predicted using bioinformatics databases (TarBase and Targetscan) in this study. In contrast to the expression of microRNA, the protein abundance of MKK6, the potential target of miR-140-3p, and TP53RK, the potential target of miR-30d-3p, were both upregulated in AS vessels and H_2_O_2_-treated endothelial cells. TP53RK is an upstream kinase of p53. It phosphorylates (serine residue Ser15) and mediates p53 activity (31). MKK6 is a protein kinase that specifically activates p38 (32). p53 and MKK6/p38 stress cascade play crucial roles in endothelial injury associated with hyperglycemia, hyperlipidemia, ischemia, etc. (33–35). In addition, we analyzed the signaling pathways that might be annotated or regulated by these target genes, among which the most significant signal pathways are MAPK and PI3K-AKT signaling pathway. Related studies have confirmed that activation of p38MAPK signaling pathway in endothelial cells is involved in vascular endothelial injury in early atherosclerosis. MAPK signaling pathway in endothelial cells regulates endothelial cell permeability, apoptosis and angiogenesis (36, 37). PI3K-Akt signaling pathway has a broad regulatory effect on cell function, and is involved in aortic vascular endothelial cell injury and atherosclerotic plaque formation (38, 39). The results of this study preliminarily revealed the correlation between circRNA_ABCA1 and circRNA_KHDRBS1 and MAPK and PI3K signaling pathways, providing reference for clarifying the role of circRNAs in atherosclerosis.

In summary, this study screened and verified the circRNAs differentially expressed in atherosclerosis and oxidative injured endothelial cells. We further verified the potential microRNA and mRNA targets of these differentially expressed circRNAs, and predicted the potential ceRNA mechanism and biological functions of the differentially expressed circRNAs. This study provided new ideas and targets for future studies on the biological functions of circRNA_ABCA1 and circRNA_KHDRBS1 in regulating atherosclerosis and endothelial injury.

## Data Availability Statement

The raw data supporting the conclusions of this article will be made available by the authors, without undue reservation.

## Ethics Statement

All animal protocols were approved by the Ethic Committees of Harbin Medical University (IRB of College of Pharmacy, Harbin Medical University, No. IRB3001619) and were in line with the ARRIVE guidelines and recommendations for the care and use of experimental animals.

## Author Contributions

HL: manuscript preparation and data analysis. XL, NS, TW, JZ, SY, XS, RW, and XW: investigation. YZhao and YZhan: project administration and supervision. YZhao and YZhan: funding acquisition. All authors contributed to the article and approved the submitted version.

## Conflict of Interest

The authors declare that the research was conducted in the absence of any commercial or financial relationships that could be construed as a potential conflict of interest.
