# Supplementary material for: Differentially Expressed Circular Non-coding RNAs in Atherosclerotic Aortic Vessels and Their Potential Functions in Endothelial Injury
Source: Front Cardiovasc Med. 2021 Jul 7;8:657544. doi: 10.3389/fcvm.2021.657544 (PMC8294331; doi:10.3389/fcvm.2021.657544)
Supplement: Supplementary file 1 [file Data_Sheet_1.zip › supplementary meterials/Supplementary material 5. Primer sequence of circRNAs.docx]

**Supplementary material 5. Primer sequences of circRNAs.**

| **circRNA** | **Primer** | **Annealing temperature (℃)** | | **Product length**  **(bp)** |
| --- | --- | --- | --- | --- |
| GAPDH(MOUSE) | F:5’ CACTGAGCAAGAGAGGCCCTAT3’  R:5’ GCAGCGAACTTTATTGATGGTATT3’ | | 60 | 144 |
| mmu_circRNA_42617 | F:5’ CGACTTCTTAGAGATCAGCGGG 3’  R:5’ GGCCACCACAGTTCTGACCATA 3’ | | 60 | 60 |
| mmu_circRNA_27503 | F:5’ TCATTGCTCATGTTCTCTTGGA 3’  R:5’ CTTGTCTGTTTTCTTCACTGCC 3’ | | 60 | 65 |
| mmu_circRNA_28589 | F:5’ GACCTGATAGTGAGCATGAGAA 3’  R:5’ TATTCATTCCAGATAACCTTTG 3’ | | 60 | 108 |
| mmu_circRNA_014193 | F:5’ CCACTTCAACATTGTAATTCCG 3’  R:5’ TGGGAGTAGAGGTGAGATATCG 3’ | | 60 | 80 |
| mmu_circRNA_24705 | F:5’ GAGAATTTCAACTGTACGGTCA 3’  R:5’ CATTGTCCTCAGATCTCAGACA 3’ | | 60 | 85 |
| mmu_circRNA_35784 | F:5’ GAAGGAGGAGCTTGACCTTG 3’  R:5’ GCCATTTGCCACCTCTATC 3’ | | 60 | 82 |
| mmu_circRNA_35619 | F:5’ AACCAGCAACCAGCACATGAA 3’  R:5’ AGCTTCTCAAAGTTGACCAGTCC 3’ | | 60 | 61 |
| mmu_circRNA_39714 | F:5’ ACACCCTTCAGCACGGGTT 3’  R:5’ CCCATCTTGACATTCTTCCCA 3’ | | 60 | 91 |
| mmu_circRNA_36781 | F:5’ CTGTGGTCTACCGAGGAAGAAG 3’  R:5’ CTGTGGCTGGTCATTAACACAA 3’ | | 60 | 96 |
| mmu_circRNA_37699 | F:5’ TTATCGCTCAGTTGCTACCC 3’  R:5’ CTTTTTTTGACTCTCCCTTCTG 3’ | | 60 | 66 |
