# Supplementary material for: Differentially Expressed Circular Non-coding RNAs in Atherosclerotic Aortic Vessels and Their Potential Functions in Endothelial Injury
Source: Front Cardiovasc Med. 2021 Jul 7;8:657544. doi: 10.3389/fcvm.2021.657544 (PMC8294331; doi:10.3389/fcvm.2021.657544)
Supplement: Supplementary file 1 [file Data_Sheet_1.zip › supplementary meterials/Supplementary material 6. Primer sequence of miRNAs.docx]

**Supplementary Material 6. Primer sequences of miRNAs.**

| **miRNA** | **Primer** |
| --- | --- |
| U6  （Mouse） | **RT:**5’>CGCTTCACGAATTTGCGTGTCAT<3’  **F:**5’>GCTTCGGCAGCACATATACTAAAAT<3’  **R:**5’>CGCTTCACGAATTTGCGTGTCAT<3’ |
| miR-30d-3p | **RT**:5’<GTCGTATCCAGTGCGTGTCGTGGAGTCGGCAATTGCACTGGATACGACGCAGCAA>3’  **F:** 5’<GCGCCTTTCAGTCAGATGT>3’  **R:** 5’<CAGTGCGTGTCGTGGAGT>3’ |
| miR-140-3p | **RT**:5’<GTCGTATCCAGTGCGTGTCGTGGAGTCGGCAATTGCACTGGATACGACCCGTGGT>3’  **F**: 5’<GCGCTAVVACAGGGTAGA>3’  **R**: 5’<CAGTGCGTGT CGTGGAGT>3’ |
